# LB5. PROVENT: Phase 3 Study of Efficacy and Safety of AZD7442 (Tixagevimab/Cilgavimab) for Pre-exposure Prophylaxis of COVID-19 in Adults

**DOI:** 10.1093/ofid/ofab466.1646

**Published:** 2021-12-04

**Authors:** Myron J Levin, Andrew Ustianowski, Stéphane De Wit, Odile Launay, Miles Avila, Seth Seegobin, Alison Templeton, Yuan Yuan, Philip Ambery, Rosalinda H Arends, Rohini Beavon, Karen A Near, Kelly W Padilla, Konstantina Psachoulia, Audrey Sharbaugh, Katie Streicher, Menelas N Pangalos, Mark T Esser, Robert A Gasser

**Affiliations:** 1 University of Colorado Anschutz Medical Campus, Aurora, CO; 2 North Manchester General Hospital, Manchester, England, United Kingdom; 3 CHU St-Pierre, Brussels, Brussels Hoofdstedelijk Gewest, Belgium; 4 Université de Paris, Inserm F-CRIN I-REIVAC, Paris, Ile-de-France, France; 5 AstraZeneca, Gaithersburg, Maryland; 6 AstraZeneca, Gaithersburg, MD, USA, Gaithersburg, Maryland

## Abstract

**Background:**

Vaccines effectively prevent COVID-19, but some individuals have medical comorbidities or receive therapies that impair their immune response to vaccination, or are ineligible for vaccination. For such individuals who remain at risk of COVID-19, monoclonal antibodies may provide additional rapid protection. AZD7442 comprises 2 fully human extended half-life SARS-CoV-2–neutralizing antibodies that bind distinct epitopes of the viral spike protein receptor binding domain. AZD7442 is in development for the prevention and treatment of COVID-19. Here, we report primary Phase 3 study results of AZD7442 for pre-exposure prophylaxis of symptomatic COVID-19.

**Methods:**

PROVENT (NCT04625725) is a Phase 3, 2:1 randomized, double-blind, placebo-controlled study of a single 300-mg AZD7442 dose (2 intramuscular injections; 150 mg each of tixagevimab and cilgavimab) for symptomatic COVID-19 prevention. Participants were unvaccinated adults (≥ 18 years old) without prior SARS-CoV-2 infection, who may benefit from immunoprophylaxis with antibodies due to an increased risk of either inadequate response to vaccination or SARS-CoV-2 exposure. The primary study endpoints were first case of SARS-CoV-2 RT-PCR-positive symptomatic illness post dose and prior to Day 183 (efficacy), and safety of AZD7442.

**Results:**

In total, 5197 participants (mean age 53.5 years, 46% female) were randomized and dosed (safety analysis set): AZD7442 n=3460; placebo n=1737. In the primary efficacy analysis (full pre-exposure analysis set, n=5172), AZD7442 reduced the risk of developing symptomatic COVID-19 by 77% (95% confidence interval 46.0, 90.0) vs placebo (P< 0.001) (Table). Adverse events occurred in 35% and 34% of participants administered AZD7442 and placebo, respectively, and injection site reactions occurred in 2.4% and 2.1% of participants, respectively (safety analysis set). There was 1 case of severe/critical COVID-19 and 2 COVID-19–related deaths in the placebo arm.

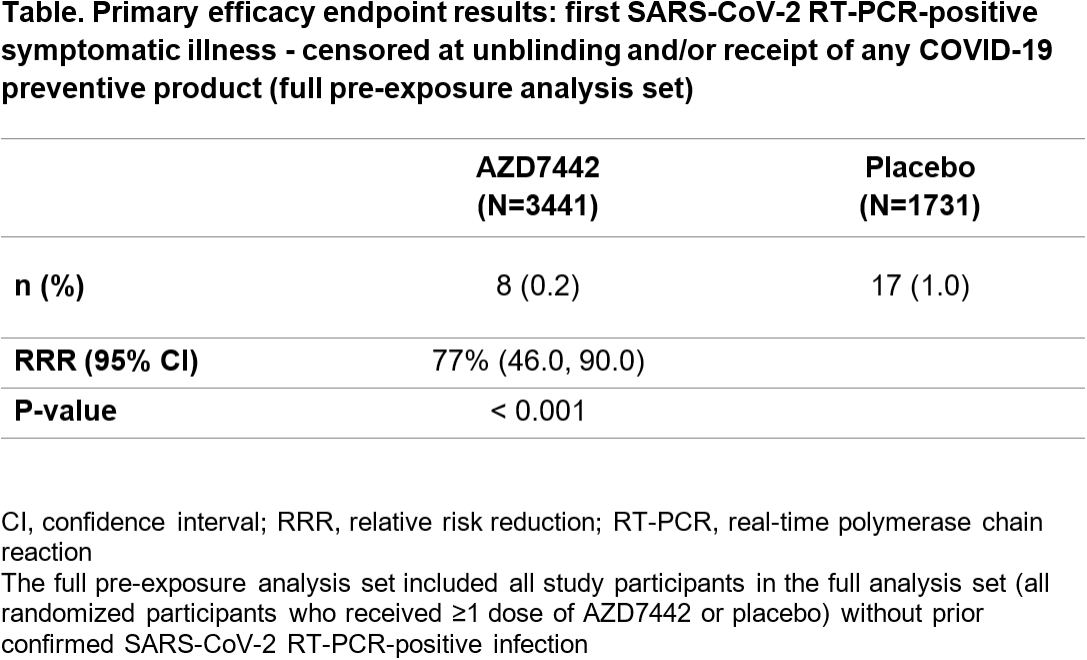

**Conclusion:**

The primary study endpoints were met: a one-time dose of AZD7442 demonstrated statistically significant protection against symptomatic COVID-19 and was well tolerated. AZD7442 is the first long-acting monoclonal antibody combination that represents a potential new option to augment COVID-19 prevention.

PROVENT funding statement image

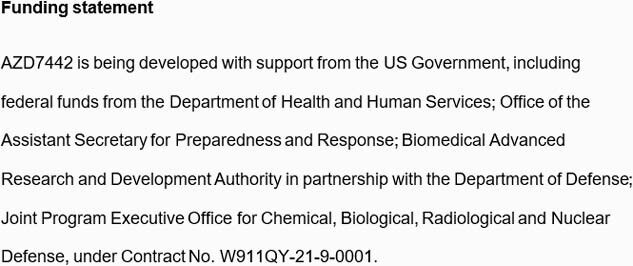

**Disclosures:**

**Myron J. Levin, MD**, **GSK group of companies** (Employee, Research Grant or Support) **Andrew Ustianowski, MBBS**, **Vir/GlaxoSmithKline** (Advisor or Review Panel member) **Stéphane De Wit, MD**, **Gilead** (Grant/Research Support)**Janssen** (Grant/Research Support)**Merck Sharpe & Dohme** (Grant/Research Support)**ViiV Healthcare** (Grant/Research Support) **Odile Launay, MD, PhD**, **AstraZeneca** (Grant/Research Support)**GlaxoSmithKline** (Consultant, Grant/Research Support, Other Financial or Material Support, Data safety monitoring board)**Johnson & Johnson** (Consultant, Grant/Research Support)**Moderna** (Consultant)**Pfizer** (Consultant, Grant/Research Support)**Sanofi Pasteur** (Consultant, Grant/Research Support) **Miles Avila, MPH, GStat**, **AstraZeneca** (Employee, Shareholder) **Seth Seegobin, PhD**, **AstraZeneca** (Employee, Shareholder) **Alison Templeton, PhD**, **AstraZeneca** (Employee, Shareholder) **Yuan Yuan, PhD**, **AstraZeneca** (Employee, Shareholder) **Philip Ambery, FRCP**, **AstraZeneca** (Employee, Shareholder) **Rosalinda H. Arends, PhD**, **AstraZeneca** (Employee, Shareholder) **Rohini Beavon, PhD**, **AstraZeneca** (Employee, Shareholder) **Karen A. Near, MD**, **AstraZeneca** (Employee, Shareholder) **Kelly W. Padilla, PharmD**, **AstraZeneca** (Employee, Shareholder) **Konstantina Psachoulia, PhD**, **AstraZeneca** (Employee, Shareholder) **Audrey Sharbaugh, PhD**, **AstraZeneca** (Employee, Shareholder) **Katie Streicher, PhD**, **AstraZeneca** (Employee, Shareholder) **Menelas N. Pangalos, PhD**, **AstraZeneca** (Employee, Shareholder) **Mark T. Esser, PhD**, **AstraZeneca** (Employee, Shareholder) **Robert A. Gasser, Jr., MD**, **AstraZeneca** (Employee, Shareholder)

